# Serum albumin and mortality risk in a hyperendemic area of HCV infection in Japan

**DOI:** 10.1186/1743-422X-7-375

**Published:** 2010-12-31

**Authors:** Yumiko Nagao, Michio Sata

**Affiliations:** 1 Department of Digestive Disease Information & Research, Kurume University School of Medicine, Kurume, Fukuoka, 830-0011, Japan; 2 Division of Gastroenterology, Department of Medicine, Kurume University School of Medicine, Kurume, Fukuoka, 830-0011, Japan

## Abstract

**Background:**

Hypoalbuminemia has been shown to be associated with increased mortality. We reported a mass screening in 1990 of X town in Japan, which demonstrated a high prevalence of hepatitis C virus (HCV) infection. This follow-up study determined, through a period of 12 years, whether serum albumin levels impact on the life prognosis of the residents of X town.

**Results:**

Of the 509 subjects, 69 had died and 55 had moved to other regions by 2002. Therefore, we analyzed 454 people for whom we could confirm life and death between 1990 and 2002. Albumin levels were assigned to two groups, low (<4.0 g/L, group A) and normal (≥4.0 g/L, group B). Of the 454 subjects analyzed, 25 were in group A and 429 in group B and the mortality was 68.0% (17/25 cases, P < 0.00001 vs. group B) and 12.1% (52/429), respectively. Mortality from hepatocellular carcinoma (HCC) was 66.7% in group A (6/9 cases, P = 0.01 vs. group B) and 15.8% (3/19) in group B. According to multivariate analysis, five factors - 50 years or older, low albumin level (<4.0 g/L), abnormal AST level, history of smoking, and absence of alcohol consumption - were associated with death. The adjusted odds ratios for these five factors were 20.65, 10.79, 2.58, 2.24 and 2.08, respectively, and each was statistically significant.

**Conclusions:**

We show that the serum albumin level is an independent risk factor for mortality from all causes in the residents of X town and an important prognostic indicator. Improvement of hypoalbuminaemia should be considered for improvement of prognosis.

## Background

Hypoalbuminemia can be caused by various conditions, including nephrotic syndrome [1,2], heart failure [3], liver disease [4,5] and malnutrition [6]. Most cases of hypoalbuminemia among hospitalized patients are caused by acute and chronic inflammatory responses [7]. Moreover, a strong association has been reported between the serum albumin level and mortality [8]. The serum albumin level is an independent risk factor for all-cause mortality in older persons and an important prognostic indicator [9].

From 1990, we have continued carrying out health screenings of the residents of X town (adult population: 7,389) in northern Kyushu, Japan, where the prevalence of hepatitis C virus (HCV) infection is the highest in the country and the mortality from liver cancer is about three times the national average [10-23]. The positive rates of antibodies to HCV (anti-HCV), HCV RNA and hepatitis B surface antigen (HBsAg) were, respectively, 23.6%, 17.9%, and 2.6% in 1990 [15]. We demonstrated extrahepatic manifestations as well as the natural course and carcinogenesis of HCV-infected persons in X town.

There has been little discussion about hypoalbuminemia and mortality over the long term in residents of the area. In this study, we determined whether serum albumin levels impact on the life prognosis of the residents of X town after a follow-up period of 12 years.

## Methods

### Subjects

In 1990, 10% (739 people) of the 7,389 inhabitants were selected randomly and, as a result, 509 subjects participated in the study for examination of liver diseases accompanying HCV or hepatitis B virus (HBV) infections [15]. We studied 509 consecutive residents prospectively for 12 years. Of these 509 subjects, 69 had died and 55 had moved to other regions by May 31, 2002. Therefore, 385 of the original inhabitants investigated in 1990 continued to reside in X town in May 2002. Consequently, 454 residents, whose life and death could be confirmed between 1990 and 2002, were studied. The albumin levels were categorized into two groups, low (<4.0 g/L, group A) and normal (≥4.0 g/L, group B) and there were 25 subjects in group A and 429 in group B.

### Serological assays

In 1990, sera were provided by the 454 subjects for the following serological assays: albumin, serum aspartate aminotransferase (AST) and alanine aminotransferase (ALT). Sera were also examined for the presence or absence of markers of HCV and HBV infection. Anti-HCV was measured by a chemiluminescent enzyme immunoassay (CLEIA) kit (Lumipulse II HCV, Fujirebio Inc., Tokyo, Japan). HCV RNA was detected in the sera using the Amplicor HCV test (Nippon Roche, Tokyo, Japan). HBsAg was assayed by a chemiluminescent immunoassay (CLIA) kit (Architect™, HBsAg QT, Dainabot Co. Ltd., Tokyo, Japan). Ultrasonographic examination of subjects with abnormalities in their liver function tests and who were positive for anti-HCV or HBsAg was performed in order to investigate the shape of the liver and lesions occupying the hepatic space.

### Physical examination

Obesity was defined as a body mass index (BMI) ≥ 25 kg/m^2 ^or greater. We also took a history of liver diseases, smoking, and alcohol consumption. We compared these factors between group A and group B. The total intake of alcohol was estimated on the basis of information about the consumption of beer, wine, whisky, Japanese sake, and shochu. In addition, the cumulative ethanol consumption up to 1990, expressed in kilograms, was calculated approximately by converting the alcohol intake in a serving of each type of alcoholic beverage into grams.

### Analysis of cause of death of the 69 individuals who had died by 2002

Of the 509 inhabitants examined in 1990, 69 (34 men and 35 women; mean age at death, 76.6 years) had died by 2002. We compared the causes of death in group A and group B.

### Statistical analysis

All data are expressed as mean ± standard error. Differences between the two groups were analyzed using the Mann-Whitney U test, Wilcoxon's test, and the Fisher's exact test. Differences were judged significant for p < 0.05 (two-tailed). Adjusted odds ratios were calculated using logistic regression analysis. All statistical analyses were conducted using JMP Version 6 (SAS Institute, Cary, NC, USA). The level of statistical significance was defined as 0.05. Survival analysis was carried out using the Kaplan-Meier method.

## Results

### Risk factors by univariate analysis

**Figure 1:**
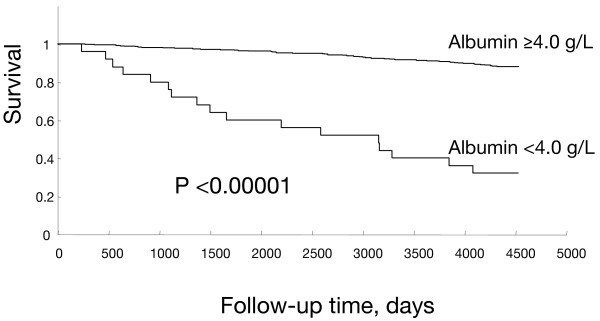
**12-year cumulative survival from 1990 to 2002 according to serum albumin concentration**. Mortality of group A (albumin < 4.0 g/L) and group B (albumin ≥ 4.0 g/L) was 68.0% (17/25 cases, P < 0.00001 vs. group B) and 12.1% (52/429), respectively.

The details of the 454 subjects studied are shown in Table 1. We compared the characteristics of 25 subjects whose serum albumin was <4.0 g/L (group A) and 429 subjects whose serum albumin was ≥4.0 g/L (group B). The mean age in group A was 68.8 ± 14.5 years and there were 16 men and nine women. The mean age in group B was 51.9 ± 15.9 years and there were 180 men and 249 women. Being male (P < 0.05), elderly (P < 0.0001), having a history of liver diseases (P < 0.01), history of smoking (P < 0.05), abnormal AST level (P < 0.01), being positive for anti-HCV (P = 0.0001), positive for HCV RNA (P < 0.001), and occurrence of death (P < 0.00001) were significantly more common in group A than in group B (Table 1). Mortality was 68.0% in group A (17/25 cases, P < 0.00001 vs. group B) and 12.1% (52/429) in group B, as shown in Table 1 and Figure 1. No significant differences were observed between the two groups regarding BMI, alcohol consumption, ALT level, and positive rate of HBsAg.

**Table 1:** Characteristics of subjects with low and normal albumin levels

	Group A		Group B		P value
	Alb < 4.0 g/L		Alb ≥ 4.0 g/L		
	n = 25		n = 429		
Age (mean ± SD), years	68.8 ± 14.5		51.9 ± 15.9		<0.0001

Sex (male/female)	16/9		180/249		<0.05

BMI ≥ 25	4	(16.0%)	54	(12.6%)	NS

History of liver diseases (yes)	15	(60.0%)	143	(33.3%)	<0.01

Alcohol consumption (yes)	13	(52.0%)	214	(49.9%)	NS

History of smoking (yes)	13	(52.0%)	139	(32.4%)	<0.05

AST (IU/L) (mean ± SD)	47.0 ± 45.8		23.6 ± 14.7		<0.01

ALT (IU/L) (mean ± SD)	44.0 ± 71.2		22.8 ± 21.4		NS

Anti-HCV, positive	14	(56.0%)	96	(22.4%)	0.0001

HCV RNA, positive	11	(44.0%)	67	(15.6%)	<0.001

HBsAg, positive	1	(4.0%)	9	(2.1%)	NS

Death by 2002	17	(68.0%)	52	(12.1%)	<0.00001

Individuals were stratified according to cumulative ethanol consumption by 1990: non-drinkers (227, 50.0%), <10 kilogram (62, 13.7%), 10-50 kilogram (37, 8.1%), 50-100 kilogram (21, 4.6%), and ≥100 kilogram (107, 23.6%).

Table 2 shows causes of death for groups A and B. The numbers of deaths from malignant tumor were 9 (52.9%) in group A and 19 (36.5%) in group B. These fatal malignant tumors were hepatocellular carcinoma (HCC, six), gastric cancer (two) and prostate cancer (one) in group A and lung cancer (six), colon cancer (four), HCC (three), gastric cancer (two), esophageal cancer (one), leukemia (one), malignant lymphoma (one) and unknown (one) in group B. Mortality from HCC was 66.7% (6/9 cases, P = 0.01 vs. group B) in group A and 15.8% (3/19) in group B. No significant differences were observed between these two groups in terms of the numbers of death from malignant tumors other than HCC.

**Table 2:** Causes of death of subjects with low and normal albumin levels

		Group A		Group B		P value
		Alb < 4.0 g/L		Alb ≥ 4.0 g/L		
		n = 17		n = 52		
Malignant tumor	HCC	6	66.7%	3	15.8%	0.01
	
	gastric cancer	2	22.2%	2	10.5%	NS
	
	prostate cancer	1	11.1%	0	0.0%	NS
	
	lung cancer	0	0.0%	6	31.6%	NS
	
	colon cancer	0	0.0%	4	21.1%	NS
	
	esophageal cancer	0	0.0%	1	5.3%	NS
	
	leukemia	0	0.0%	1	5.3%	NS
	
	malignant lymphoma	0	0.0%	1	5.3%	NS
	
	unknown	0	0.0%	1	5.3%	NS
	
	total	9	52.9%	19	36.5%	NS

Cerebrovascular disease		0	0.0%	10	19.2%	NS

Cardiac disease		3	17.6%	6	11.5%	NS

Pneumonia		3	17.6%	6	11.5%	NS

Liver disease		1	5.9%	3	5.8%	NS

Diabetes mellitus		0	0.0%	2	3.8%	NS

Suicide		0	0.0%	2	3.8%	NS

Tuberculosis		0	0.0%	1	1.9%	NS

Freak accident		1	5.9%	1	1.9%	NS

Feebleness of age		0	0.0%	1	1.9%	NS

Other		0	0.0%	1	1.9%	NS

HCC, hepatocellular carcinoma

No significant differences were observed between the two groups for mortality from cerebrovascular disease, cardiac disease, pneumonia, liver disease, diabetes mellitus, suicide, tuberculosis, a freak accident, feebleness of age, and others.

### Multivariate analysis

According to multivariate analysis, five factors - 50 years or older, low albumin level (<4.0 g/L), abnormal AST level, history of smoking, and absence of alcohol consumption - were associated with death. The adjusted odds ratios for these five factors were 20.65, 10.79, 2.58, 2.24 and 2.08, respectively, and each was statistically significant (Table 3).

**Table 3:** Results of multivariate analysis

		Adjusted odds ratio			P value
			
		(95% confidence interval)			
50 years or older	20.65	7.08	-	88.71	<0.0001

Albumin < 4.0 g/L	10.79	4.02	-	32.75	<0.0001

Abnormal AST level (≥40 IU/L)	2.58	1.14	-	5.79	<0.05

History of smoking (yes)	2.24	1.08	-	4.65	<0.05

Non-alcohol consumption	2.08	1.03	-	4.36	<0.05

Cumulative ethanol consumption of <10 kilogram or 10-50 kilogram played an important role in survival. The adjusted odds ratios compared to absence of alcohol consumption were 6.44 (95% confidence interval: 1.93-39.92), and 7.72 (95% confidence interval: 1.62-138.46), respectively.

## Discussion

Low serum albumin levels are an important predictor of morbidity and mortality [8,9] and correlate with an increased risk of morbidity and mortality in hospitalized patients. However, there has been little discussion about hypoalbuminemia and mortality of the residents of an area with an exceptionally high prevalence of HCV infection. In this study, we determined whether serum albumin levels affect the life prognosis of the residents of X town.

Our results indicate a strong association between hypoalbuminemia and mortality in this hyperendemic area of HCV infection in Japan. Residents with hypoalbuminemia had a mortality of 68.0%; dramatically higher than the rate of 12.1% among residents who had normal albumin levels. We previously reported that HCV infection and ALT value were associated with deaths due to HCC or liver cirrhosis in this X town [17]. We also showed that hypoalbuminemia was prognostic factor about all-cause mortality.

It is estimated that ~170 million people worldwide are infected with HCV [24], some two million (1%) of whom reside in Japan [25]. HCV leads to serious consequences such as liver cirrhosis and HCC. Of the HCC cases in Japan, around 16% are caused by hepatitis B virus (HBV) infection and around 80% by HCV infection. The increase in the number of HCC patients due to HCV contributes to the increase in total deaths in Japan from HCC. This trend is expected to continue until 2015 [25].

Albumin, produced only by the liver, is the major protein that circulates in the blood. Albumin consists of 585 amino acids, has a molecular weight of approximately 69 kDa and is the most abundant plasma protein, although 60% of the total albumin pool is in the interstitial space [26]. Albumin is essential for maintaining the oncotic pressure in the vascular system. A decrease in oncotic pressure due to a low albumin level allows fluid to leak from the interstitial spaces into the peritoneal cavity, producing ascites. Albumin is also very important in the transportation of various molecules, including bilirubin, free fatty acids, drugs, and hormones. Serum albumin is an abundant multifunctional non-glycosylated, negatively charged plasma protein, with ascribed ligand-binding and transport properties, antioxidant functions, and enzymatic activities [27].

A low serum albumin concentration indicates poor liver function. Decreased serum albumin levels are not seen in acute liver failure because it takes several weeks of impaired albumin production until the serum albumin level drops. The most common reason for a low albumin is chronic liver failure caused by cirrhosis. The serum albumin concentration is usually normal in chronic liver disease, until cirrhosis and significant liver damage develops. In advanced liver disease, the serum albumin level may be less than 3.5 g/dl. The albumin level is clinically important as a predictive factor for patients with liver cirrhosis, because decreased serum albumin levels cause ascites and edema.

Recent studies have demonstrated the efficacy of branched-chain amino acid (BCAA) supplementation in improving hypoalbuminemia in cirrhotic patients [28]. Kotho et al. investigated the correlation between albumin levels and the fat-free mass in cirrhotic patients [29]. They showed that exercise and protein-rich nutrition at the early stage of liver cirrhosis may be advisable for maintaining or increasing muscular volume. Nishiguchi et al reported that if cirrhotic patients were in the compensated stage at the entry but with lower BCAA tyrosine ratio (BTR), oral BCAA supplementation might be effective in maintaining serum albumin [30]. Stating appropriate nutritional interventions, such as supplementation of BCAA, in the early stage of cirrhosis may improve prognosis and maintain QOL. We also reported that the administration of BCAA supplement (Aminofeel^®^) increases serum albumin levels and serum zinc levels, and improves sensitivity to different tastes [31-33].

## Conclusions

In conclusion, we demonstrated that the serum albumin level is an independent risk factor for mortality from all causes and an important prognostic indicator in the residents of X town. In particular, improvement of hypoalbuminaemia as well as the eradication of HCV, such as by interferon therapy, should be considered for improvement of prognosis in this hyperendemic area of HCV infection in Japan.

## Abbreviations

HBV: hepatitis B virus; HBsAg: hepatitis B surface antigen; HCV: hepatitis C virus; anti-HCV: anti-bodies to HCV; HCC: hepatocellular carcinoma; CLEIA: chemiluminescent enzyme immunoassay; BCAA: branched-chain amino acids

## Competing interests

The authors declare that they have no competing interests.

## Authors' contributions

YN carried out most of the data collection and drafted the manuscript. MS contributed to data analysis. All authors read and approved the final manuscript.
